# Obstructive Sleep Apnea is Common and Associated with Heart Remodeling in Patients with Chagas Disease

**DOI:** 10.5935/abc.20180131

**Published:** 2018-09

**Authors:** Carolina de Araújo Medeiros, Isaac Vieira Secundo, Carlos Antônio da Mota Silveira, José Maria del Castilho, Afonso Luiz Tavares de Albuquerque, Sílvia Marinho Martins, Wilson de Oliveira Júnior, Geraldo Lorenzi-Filho, Luciano F. Drager, Rodrigo Pinto Pedrosa

**Affiliations:** 1 Laboratório do Sono e Coração do Pronto Socorro Cardiológico de Pernambuco (PROCAPE) da Universidade de Pernambuco, Recife, PE - Brazil; 2 Ambulatório de Doença de Chagas e insuficiência Cardíaca - PROCAPE da Universidade de Pernambuco, Recife, PE - Brazil; 3 Instituto do Coração (InCor) - Hospital das Clínicas da Faculdade de Medicina da Universidade de São Paulo (HCFMUSP), São Paulo, SP - Brazil

**Keywords:** Chagas Disease, Sleep Apnea, Obstructive, Ventricular Remodeling, Arrhythmias, Cardiac

## Abstract

**Background:**

Chagas Disease (CD) is an important cause of morbimortality due to heart failure and malignant arrhythmias worldwide, especially in Latin America.

**Objective:**

To investigate the association of obstructive sleep apnea (OSA) with heart remodeling and cardiac arrhythmias in patients CD.

**Methods:**

Consecutive patients with CD, aged between 30 to 65 years old were enrolled. Participants underwent clinical evaluation, sleep study, 24-hour Holter monitoring, echocardiogram and ambulatory blood pressure monitoring.

**Results:**

We evaluated 135 patients [age: 56 (45-62) years; 30% men; BMI: 26 ± 4 kg/m^2^, Chagas cardiomyopathy: 70%]. Moderate to severe OSA (apnea-hypopnea index, AHI, ≥ 15 events/h) was present in 21% of the patients. OSA was not associated with arrhythmias in this population. As compared to patients with mild or no OSA, patients with moderate to severe OSA had higher frequency of hypertension (79% vs. 72% vs. 44%, p < 0.01) higher nocturnal systolic blood pressure: 119 ± 17 vs. 113 ± 13 vs. 110 ± 11 mmHg, p = 0.01; larger left atrial diameter [37 (33-42) vs. 35 (33-39) vs. 33 (30-36) mm, p < 0.01]; and a greater proportion of left ventricular dysfunction [LVEF < 50% (39% vs. 28% vs. 11%), p < 0.01], respectively. Predictor of left atrial dimension was Log_10_ (AHI) (β = 3.86, 95% CI: 1.91 to 5.81; p < 0.01). Predictors of ventricular dysfunction were AHI > 15 events/h (OR = 3.61, 95% CI: 1.31 - 9.98; p = 0.01), systolic blood pressure (OR = 1.06, 95% CI: 1.02 - 1.10; p < 0.01) and male gender (OR = 3.24, 95% CI: 1.31 - 8.01; p = 0.01).

**Conclusions:**

OSA is independently associated with atrial and ventricular remodeling in patients with CD.

## Introduction

Chagas disease (CD) is the third most common parasitic infection, after malaria and schistosomiasis, affecting about 7 to 8 million people worldwide.^1^ CD is caused by the protozoan *Trypanosoma cruzi,* transmitted to humans by insects (*Triatominae*), blood transfusion, organ and tissue transplantation, oral contamination or congenital transmission.^2^ The epidemiological profile of the disease has been modified in recent decades, due to migratory flows,^2^ thus also generating attention in non-endemic countries such as the United States, Canada, Spain, Italy and Japan.^3,4^

Chagas cardiomyopathy is the most common form of nonischemic cardiomyopathy and one of the leading causes of complications and death in Latin America.^5^ Approximately one third of patients with CD develop Chagas cardiomyopathy^3^ characterized by ventricular arrhythmias, cardiac blockages, alterations in cardiac proteins with heart remodeling, heart failure and sudden death. Heart failure due to CD worsens patient prognosis, when compared with other cardiomyopathies.^6^ In addition to the myriad of characteristics involved in CD, it is important to consider potential comorbidities that may have a negative impact on patients' health.

Obstructive sleep apnea (OSA) is the most frequent respiratory disturbance in the overall population and is associated with heart remodeling and arrhythmias in patients without^7,8^ and with comorbidities, including heart failure.^6^ However, whether this association exists in patients with CD is unknown. We hypothesized that OSA is independently associated with cardiac arrhythmias and heart remodeling in patients with CD.

## Methods

### Subjects

Consecutive patients with CD (with two positive blood tests-immunofluorescence and ELISA), aged between 30 and 65 years old were recruited from a specialized outpatient service from August 2013 to August 2014. Chagas cardiomyopathy was diagnosed in patients with serological evidence of antibodies to *T. cruzi* and evidence of Chagas heart disease who may or may not have cardiac symptoms (such as dyspnea, edema, and chest pain). The indeterminate form of CD was diagnosed in patients with serological and/or parasitological evidence of *T. cruzi* infection who lacked symptoms, physical signs, electrocardiographic abnormalities and any radiographic evidence (on chest radiography, barium-contrast esophageal, or colon radiography) of cardiac or gastrointestinal involvement.^9^ Patients with cardiac pacemakers, manifest or suspected coronary disease; decompensated heart failure requiring hospital admission, predominant central sleep apnea (>50% of events scored as central), or renal disease (serum creatinine >2mg/dL), as well as those with a previous stroke were excluded.

Patients were invited to undergo the sleep study in the week after they underwent all examinations, including echocardiography, Ambulatory Blood Pressure Monitoring (ABPM) and 24-hour Holter monitoring as described below.

### Sleep Study

All patients underwent portable sleep monitoring in the sleep laboratory using a validated device (Embletta Gold, PDS; Medcare, Reykjavik, Iceland)^10^ to evaluate oxygen saturation, body position, nasal flow measurements (pressure cannula), and respiratory effort measurements using two respiratory inductance plethysmography belts. All exams were scored by an experienced physician. Apnea was defined as total absence (>90%) and hypopneas as a decrease (>30%) in nasal flow for ≥ 10 seconds, followed by a 4% desaturation (in hypopneas), respectively.^11^ The apnea-hypopnea index (AHI) was calculated by dividing the total number of apnea and hypopnea events by the total hours in bed.^11,12^

Mild OSA was defined by an AHI between 5 and 14.9 events/h and moderate to severe OSA was considered when the AHI was ≥ 15 events/h. The oxygen desaturation index (ODI) was calculated as the total number of desaturations, divided by the total time in bed.

Excessive daytime sleepiness was evaluated using the Epworth Sleepiness Scale. A total score > 10 was considered excessive daytime somnolence.^13^

### Office blood pressure

Blood pressure (BP) was measured after 5 min of rest using standard protocols.^14^ The average of two readings was obtained at 5 min intervals with an automatic digital sphygmomanometer (Omron BP742).

### Ambulatory Blood Pressure Monitoring (ABPM)

All participants underwent blood pressure monitoring for 24 hours, using SpaceLabs equipment (model 90207; SpaceLabs, Redmond, WA). The BP reading was taken every 10 minutes during the day and every 20 minutes at night, using an appropriate cuff placed on the non-dominant arm. Participants were instructed to perform their ordinary daily activities and not to move their arm during the ongoing measurement. Activity, bedtime, and time on awakening from sleep were recorded by participants in diaries.^15^ The normal BP dip was defined separately for systolic and diastolic BP as a ≥ 10% reduction in BP during sleep compared with the awake period. Nondipping was defined as a decrease of < 10%.

### Holter monitoring

Holter monitoring (Cardios®, Cardio Systems, São Paulo, Brazil) was performed in all patients for 24 hours. The following characteristics of the ECG were analyzed: baseline heart rhythm, heart rate, ventricular and atrial arrhythmias, and breaks. The complexities of the arrhythmias were described as follows: isolated, paired, or tachycardia.^16^ Patients were instructed to keep a diary with their symptoms during the exam. The Holter analysis was performed by an experienced observer, who was blinded to the presence or absence of OSA.

### Echocardiogram

A transthoracic echocardiogram was performed using a Philips IE33 S5-1 device. Conventional M-mode echocardiography was used to measure cavity dimensions (diastolic and systolic diameters, wall thickness, and aorta and left atrial size).^17^ Left atrial volumes were indexed by body surface area according to the American Society of Echocardiography.^18^ Using two-dimensional echocardiography, segmental and global contractility were assessed, and the left ventricular ejection fraction (LVEF) was calculated using Simpson's formula. Ventricular dysfunction was considered when LVEF <50%.^18^ **Le**ft ventricular longitudinal strain with speckle-tracking was calculated and values below -16% were considered abnormal.^19^ The Echocardiographic evaluation was performed by the same experienced observer, who was blinded to the presence or absence of OSA.

### Statistical analysis

Normality distribution was evaluated with the Kolmogorov-Smirnov test.

For the categorical variables, the Chi-square test of Pearson was used. Quantitative variables with a normal distribution were presented as mean and standard deviation and the ANOVA test was used, whereas the variables without normal distribution were presented as median and percentiles (P25; P75) and the Kruskal-Wallis was used, with Bonferroni post-hoc test, when appropriate.

A multiple linear regression analysis was performed to evaluate independent predictors of left atrial dimensions. The independent variables of the left atrial dimensions were age, 24-hour systolic BP, body mass index (BMI), and AIH. Due to the non-normality of the AHI, a log-transformed version of this variable was used in the multivariate model. To analyze the predictors of ventricular dysfunction, a multiple logistic regression analysis was performed with the following variables: age, BMI, male gender, 24-hour systolic BP, diabetes mellitus diagnosis, AHI ≥15 events/h, ODI, and saturation < 90%. The data were analyzed with SPSS 21.0 statistical software (IBM Corporation) and a value of p < 0.05 was considered significant.

## Results

**Figura 1:**
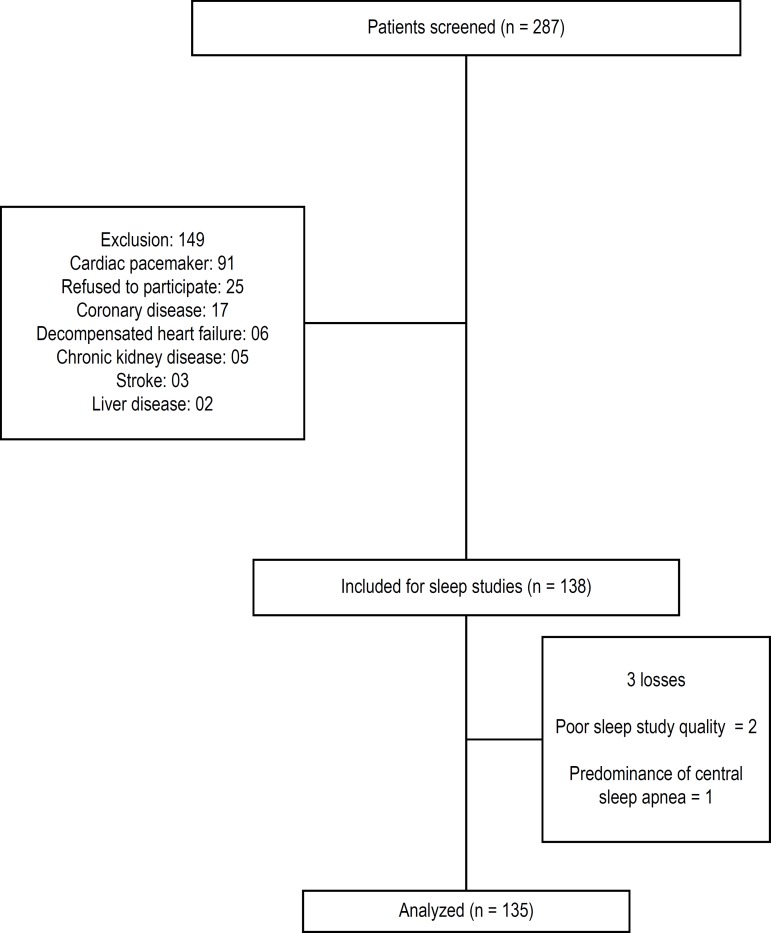
Diagrama de fluxo do paciente.

We consecutively evaluated 287 patients with CD (41 [30%] with the indeterminate form of the disease and 94 [70%] with Chagas cardiomyopathy). Most of the exclusions were due to the presence of cardiac pacemakers (Figure 1). One patient had a predominance of central sleep apnea and was also excluded, resulting in a final sample of 135 patients (Figure 1).

The frequency of OSA in patients with CD was 58%. Mild OSA was diagnosed in 50 patients (37%) and moderate-to-severe OSA (IAH ≥ 15 events/h) in 28 patients (21%). None of the participants had a previous OSA diagnosis. Patients with moderate-to-severe OSA had larger neck and waist circumferences, a higher frequency of high blood pressure and a higher percentage of them were on diuretics, b-blockers and ACE inhibitors, as well as AT1 inhibitors compared to patients with mild and no OSA, respectively (Table 1).There was no difference in the degree of sleepiness (Epworth Sleepiness Scale) between the groups (Table 1). The demographics and clinical characteristics according with the absence or presence of OSA are described in Table 1.

**Table 1:** Anthropometrics and clinical characteristics

	Total n = 135	No OSA n = 57	Mild OSA n = 50	Mod/Severe OSA n = 28	p
Age, years	56 (45-62)	48 (42-58)	59 (55-63)	61 (51-63)	< 0.001*
Male, n (%)	40 (30)	15 (26)	15 (30)	10 (36)	0.67^†^
BMI, kg/m^2^	26.3 ± 4.2	24.7 ± 3.9	27.4 ± 3.9	27.6 ± 4.5	0.001^¥^
Neck circumference, cm	35 (33-38)	34 (32-36)	36 (33-39)	38 (34-39)	0.02^†^
Waist, cm	92 (83-96)	87 (79-92)	96 (89-102)	96 (91-104)	< 0.001*
Caucasians, n (%)	43 (32)	19 (33)	16 (32%)	8 (29%)	0.86^†^
Diabetes mellitus, n (%)	17 (13)	5 (9)	7 (14)	5 (18)	0.46^†^
Hypertension, n (%)	83 (62)	25 (44)	36 (72)	22 (79)	0.001^†^
Office Systolic BP, mmHg	131 ± 22	128 ± 20	135 ± 22	133 ± 26	0.27^¥^
Office Diastolic BP, mmHg	81 ± 11	83 ± 11	81 ± 12	78 ± 11	0.14^¥^
Use of antihypertensive drugs, n	1 (0-2)	1 (0-2)	2 (1-2)	2 (0-3)	0.001^†^
Diuretics, n (%)	54 (40)	13 (23%)	26 (52%)	15 (54%)	< 0.001^†^
ACE inhibitors / AT1 inhibitors n (%)	76 (56)	23 (30%)	35 (46%)	14 (24%)	< 0.001^†^
B-blockers, n (%)	57 (42)	17 (30%)	25 (50%)	13 (54%)	0.04^†^
Spironolactone, n (%)	14 (10)	5 (9%)	5 (10%)	4 (14%)	0.73^†^
Epworth sleepiness scale, Score, n (%)	9.8 ± 4.9	9.3 ± 5.2	10.2 ± 5.2	10.1 ± 3.8	0.57^¥^

Values are mean (±SD). Variables with skewed distribution presented as median (interquartile range). Abbreviations: BMI body mass index; BP: blood pressure.

* Test of Kruskal-Wallis;

† Chi-square test of Pearson;

¥ Anova test.

Overall, there were no clinically significant differences in supraventricular and ventricular arrhythmia frequencies across the three groups. However, there was a greater proportion of supraventricular paired in patients with moderate-to-severe OSA, compared with patients with mild and no OSA (50% vs 40% and 23%; p = 0.03), respectively. (Table 2)

**Table 2:** Holter evaluation - Distribution of atrial and ventricular arrhythmias

Variable	Total n = 135	No OSA n = 57	Mild OSA n = 50	Mod/Severe OSA n = 28	p
HR Minimum (b.p.m)	50 (45-55)	50 (45-56)	50 (45-56)	52 (46-54)	0.97*
HR Average (b.p.m)	71 (65-77)	71 (66-79)	71 (65-74)	71 (62-75)	0.82*
HR Maximum (b.p.m)	113 (105-25)	118 (109-132)	112 (103-121)	109 (98-121)	0.045*
Total awake ventricular (%)	94 (70)	39 (68)	37 (74)	18 (64)	0.65^†^
Total sleep ventricular (%)	95 (70)	36 (63)	40 (80)	19 (68)	0.16^†^
Isolated ventricular arrhythmias (%)	107 (79)	44 (77)	40 (80)	23 (82)	0.86^†^
Ventricular bigeminy (%)	38 (79)	14 (25)	14 (28)	10 (36)	0.56^†^
Ventricular paired (%)	50 (37)	18 (32)	19 (38)	13 (46)	0.41^†^
Non-sustained ventricular tachycardia	27 (20)	10 (18)	10 (20)	7 (25)	0.72^†^
Total awake supraventricular	115 (85)	47 (83)	42 (84)	26 (93)	0.43^†^
Total sleep supraventricular	118 (87)	48 (84)	44 (88)	26 (93)	0.52^†^
Isolated supraventricular tachycardia (%)	124 (92)	51 (90)	48 (96)	25 (89)	0.40^†^
Supraventricular paired (%)	47 (35)	13 (23)	20 (40)	14 (50)	0.03^†^
Non-sustained supraventricular tachycardia (%)	27 (20)	10 (18)	10 (20)	7 (25)	0.26^†^
Chronic atrial fibrillation (%)	1 (0.7)	0	1 (2)	0	0.43^†^
Paroxysmal atrial fibrillation (%)	1 (0.7)	0	0	1 (3.6)	0.15^†^
Right bundle-branch block (%)	62 (46)	23 (46)	23 (54)	9 (41)	0.59^†^

HR: Heart Rate;

* Test of Kruskal-Wallis;

† Chi-square test of Pearson.

**Figura 2:**
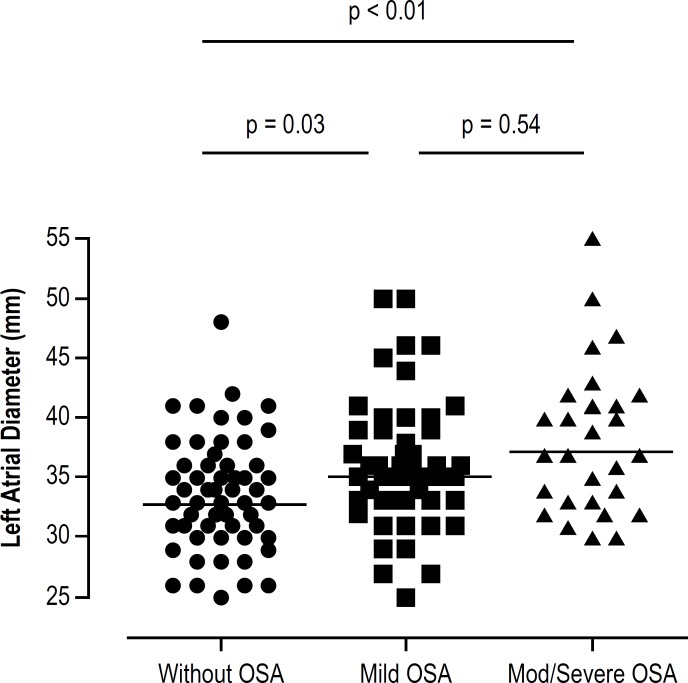
Diâmetro do átrio esquerdo em pacientes com doença de Chagas. AOS: apneia obstrutiva do sono. Teste de Kruskal-Wallis, com post-hoc de Bonferroni.

Patients with moderate-to-severe OSA had increased nocturnal blood pressure (119 ± 17 vs. 113 ± 13 and 110 ± 11 mmHg; p = 0.01) compared to patients with mild and no OSA, respectively (Table 3). The aorta size, left ventricular systolic and diastolic diameters, septum and posterior wall thickness were similar between groups. Left atrial diameter [37 (33-42) vs. 35 (33-39) and 33 (30-36) mm, p < 0.01(Figure 2) and volume [66(54-95)vs. 46(39-65) and 42(35-56) mL/m^2^, p < 0.001] were larger in patients with moderate-to-severe OSA, compared with mild and no OSA groups, respectively(Table 3).

**Table 3:** Echocardiography, Ambulatory Blood Pressure Monitoring and sleep study characteristics

Variable	Total n = 135	No OSA n = 57	Mild OSA n = 50	Mod/Severe OSA n = 28	p
**Echocardiography**					
LVFE (%)	60 (51-65)	61 (57-66)	59 (47-65)	57 (43-63)	0.09*
LA volume index (mL/m^2^)*	29 (23-38)	27 (21-33)	30 (23-38)	37 (51-55)	< 0.001*
Aorta (mm)	30 (28-33)	30 (28-32)	30 (29-34)	30 (27-34)	0.21*
LV diastolic dimension (mm)	52 (49-58)	51 (48-55)	53 (49-58)	56 (50-61)	0.24*
LV systolic dimension (mm)	34 (30-41)	32 (30-37)	34 (30-40)	39 (30-48)	0.14*
Septum (mm)	8.0 (8.0-9.0)	8.0 (7.5-9.0)	8.0 (8.0-9.0)	8.0 (8.0-9.8)	0.10*
Posterior wall thickness (mm)	8.0 (7.0-9.0)	8.0 (7.0-9.0)	8.0 (8.0-9.0)	8.0 (8.0-9.0)	0.27*
LV longitudinal strain (%)**	-17 (-20/-13)	-19 (-21/-15)	-17 (-20/-12)	-16 (-19/-13)	0.04*
**ABPM**					
Systolic BP awake, mmHg	121 ± 12	118 ± 12	121 ± 12	123 ± 14	0.22^¥^
Diastolic BP awake, mmHg	74 ± 8	76 ± 8	73 ± 8	73 ± 9	0.34^¥^
Systolic BP Sleep, mmHg	113 ± 14	110 ± 11	113 ± 13	119 ± 17	0.01^¥^
Diastolic BP sleep, mmHg	67 ± 9	67 ± 8	66 ± 9	69 ± 9	0.41^¥^
Systolic non-dipping, %	78	75	79	89	0.29^†^
Diastolic non-dipping, %	48	40	41	75	< 0.01^†^
**Sleep variables**					
AHI. Events/hour	5.8 (2.7-11.9)	2.4 (1.6-3.3)	7.2 (5.8-10.0)	20.3 (16.7-29.1)	< 0.001^¥^
Mean SaO_2_, %	96 (95-97)	97 (94-97)	96 (94-97)	95 (94-97)	< 0.001*
Lowest SaO_2_, %	88 (83-92)	92 (89-94)	86 (83-89)	83 (79-87)	< 0.001*
Desaturation index. Number/hour	5.5 (2.2-12.8)	2.1 (0.9-3.4)	7.5 (4.9-10.4)	19.0 (5.9-26.5)	< 0.001*
SaO_2_ < 90. % of night time	0 (0-3)	0 (0-0)	1 (0-4)	2 (1-5)	< 0.001*

Values are mean (±SD). Variables with skewed distribution presented as median (interquartile range). Abbreviations: LVFE: left ventricular ejection fraction; LA: left atrial; LV: left ventricular; ABPM: ambulatory blood pressure measurement; BP: blood pressure; AHI: apnoea-hypopnoea index; SaO_2_: arterial oxyhemoglobin saturation.

** Data from 103 patients.

* Test of Kruskal-Wallis;

† Chi-square test of Pearson;

¥ Anova test.

**Figura 3:**
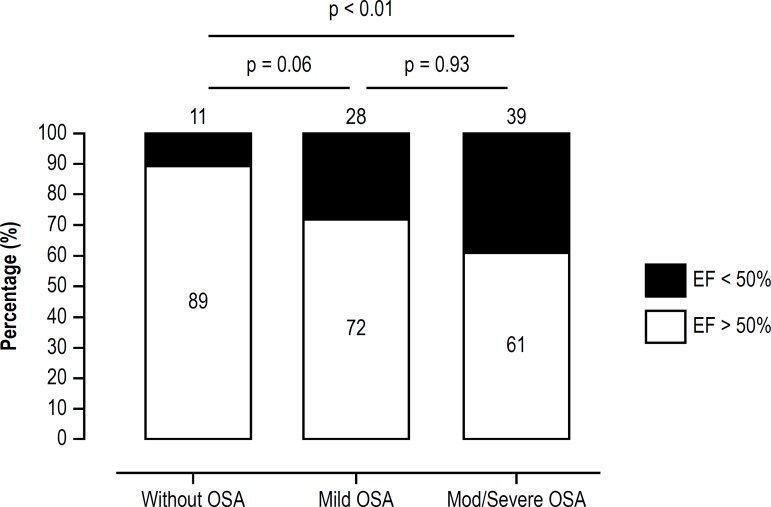
Freqüência de Apneia Obstrutiva do Sono em Pacientes com Doença de Chagas. AOS: Apneia Obstrutiva do Sono; IAH: índice de apneia-hipopneia; e/h: eventos por hora. Teste de Kruskal-Wallis.

These findings were significant when the whole study population was analyzed, as well as in the subgroup of patients with Chagas cardiomyopathy, but not in patients from the indeterminate group. Left ventricular longitudinal strain was lower in patients with moderate-to-severe OSA compared with patients with mild and no OSA -16(-19/-13)% vs. -17(-20/-12)% and -19 (-21/-15)%, p = 0.04. The prevalence of ventricular dysfunction [LVEF < 50% (39% vs 28% and 11%), p < 0.01] was also higher in the group with moderate-to-severe OSA than in participants with mild or no OSA (Figure 3). This higher prevalence was also observed in the subgroup of patients with Chagas cardiomyopathy.

The only predictor of left atrial dimension in the multivariate analysis was Log_*10*_ (AHI) (β = 3.86, 95% CI: 1.91 to 5.81; p < 0.01) in the whole population (Table 4), as well as in the subgroup with Chagas cardiomyopathy.

**Table 4:** Univariate and multiple linear regression predictors of left atrial dimension

Univariate	Multivariate				
Variable	(β)	p	(β)	CI	p
Age (Years)	0.05	0.36			
24h Systolic BP (mmHg)	-0.03	0.46			
BMI. kg/m^2^	0.32	0.01	0.16	-0.01 to 0.44	0.06
Log_10_AHI (events/hour)	3.86	< 0.001	3.86	1.91 to 5.81	< 0.01

BP: blood pressure; BMI: body mass index; AHI: Apnoea-hypopnoea; CI: Confidence interval

The independent predictors of the presence of ventricular dysfunction were AHI (≥ 15 events/h) (OR = 3.61, 95% C.I.:1.31 to 9.98; p = 0.01), systolic blood pressure (OR = 1.06, 95% C.I.: 1.02 - 1.10; p =< 0.01) and male gender (OR = 3.24, 95% C.I.: 1.31 - 8.01; p = 0.01) (Table 5).

**Table 5:** Logistic regression to assess predictors of ventricular dysfunction

	Univariate		Multivariate	
Variable	p	OR	CI	p
Age (years)	0.49			
BMI. kg/m^2^	0.09			
Male %	0.01	3.24	1.31 - 8.01	0.01
24h Systolic BP (mmHg)	0.01	1.06	1.02 - 1.10	< 0.01
DM	0.95			
AHI (≥ 15 events per hour)	0.02	3.61	1.31 - 9.98	0.01
Desaturation index. number/hour	0.25			
SaO_2_ < 90%. % of night time	0.21			

BMI: body mass index; BP: blood pressure; DM: Diabetes mellitus; AHI: apnoea-hypopnoea; OR: odds ratio; CI: Confidence interval.

## Discussion

To the best of our knowledge, this is the first study that evaluated OSA in consecutive patients with CD. We found a high frequency of moderate to severe OSA in patients with CD (21%). None of the participants had a previous diagnosis of OSA, which suggests a low awareness of the disease in this population. Patients with moderate to severe OSA were more hypertensive, used more antihypertensive drugs and had increased nocturnal blood pressure. In addition, moderate to severe OSA was independently associated with cardiac remodeling parameters, including larger left atrium dimension and a higher prevalence of ventricular dysfunction, especially in the subgroup of patients with Chagas cardiomyopathy. Taken together, our study emphasizes the concept that OSA may contribute to a poor prognosis in patients with CD.

OSA is a prevalent condition in patients with cardiovascular disease,^20^ and our study confirms its high frequency in patients with CD. Particular characteristics of studied OSA patients deserves some comments: First, the presence of higher BMI and neck circumference in patients with moderate to severe OSA as compared to patients with mild or no OSA denotes a typical phenotype of the disease (Table1). However, the lack of excessive daytime sleepiness, a characteristic of OSA patients from the overall population referred to sleep laboratories may explain the low awareness of OSA in patients with cardiovascular disease.^21^ This finding is in line with other populations with cardiovascular diseases, including heart failure^22^ and hypertrophic cardiomyopathy.^23^ The low prevalence of central sleep apnea in our sample is in contrast with a previous study that evaluated sleep disorders in patients with heart failure (LVEF < 45%), including CD. Silva et al.^24^ included 24 patients with CD, of which only 12% had OSA, but 44% of whom had Cheyne-Stokes respiration. The median LVEF of our sample was within the normal range (60%) and the majority of our sample did not have heart failure. Moreover, we excluded patients with decompensated heart failure from our study, which may explain the different frequencies.

OSA is recognized as being a cause of hypertension.^25^ In our sample, hypertension was more frequent in the group with moderate to severe OSA and this group used more antihypertensive drugs. However, only systolic BP during sleep was higher in patients with moderate-to-severe OSA. This finding may be related to the fact that the study took place in a reference center for cardiology, where most patients are being properly treated for hypertension. The relatively low frequency of atrial and ventricular arrhythmias in our study may be also explained by the exclusion of more severe cases of CD cardiomyopathy.

Our study demonstrated that in patients with CD, OSA is independently associated with left atrial enlargement. This finding is consistent with other OSA populations^7,23,26^ Rossi et al.^27^ conducted a meta-analysis of 1,157 patients who took part in 18 heart failure studies, and concluded that left atrial enlargement was associated with a worse prognosis, regardless of age, functional class, ejection fraction or diastolic function pattern,^28^ reinforcing the importance of our findings. In our study, moderate to severe OSA was also associated with a lower left ventricular longitudinal strain and a four-fold higher proportion of ventricular dysfunction than patients with no OSA. This finding is consonant with an earlier study that evaluated patients referred to a sleep laboratory and may impact negatively on mortality.^6,29^

There are several mechanisms that can contribute to cardiac remodeling in OSA patients^30^ One possible explanation for the heart remodeling in our study may be partially explained by the increased frequency of hypertension and higher nocturnal systolic blood pressure, as well as increased frequency of abnormal blood pressure dip in the moderate to severe OSA group. Taken together, the increased hypertension burden may increase arterial stiffness and left ventricular afterload, contributing to these abnormalities.^7^ Moreover, inspiratory efforts during the apneas generate negative intrathoracic pressure, which leads to an increase in the left ventricular afterload and a decrease in the left ventricular preload, which in turn cause a reduction in the ejection volume and may induce left atrial enlargement, as demonstrated in our study. Intermittent hypoxia may also influence cardiac contractility, directly or indirectly, thereby reducing cardiac output.^22^ OSA induces hypoxia, hypercapnia and sleep arousals, thus promoting an increase in sympathetic activity and hence, in blood pressure.^31^ Long-term exposure to high sympathetic nerve activity can induce hypertrophy and apoptosis of the cardiac myocytes^32^ and thereby cause left ventricular dysfunction. These adverse hemodynamic effects may be more pronounced in individuals with heart failure,^33^ as shown in our subgroup with Chagas cardiomyopathy compared with patients with the indeterminate form.

The major strength of the present study is that it is the largest and one of the only cohorts evaluating the association between sleep apnea in patients with CD. The recruitment of consecutive patients with well-characterized CD not referred to a sleep laboratory may generalizes the findings of our study, as does the use of gold-standard techniques to assess blood pressure (ABPM) and respiratory effort (inductance plethysmography belts).^11^ The study has some potential limitations. A portable sleep monitor that does not measure sleep duration was used. Thus, measurements of the AHI were taken based on the total recording time and not on the total length of sleep, although this device has already been validated against full polysomnography.^34^ Furthermore, our findings are derived from a cross-sectional study and we cannot infer causality, but only an independent association between OSA and heart remodeling. The fact that we could not demonstrate the same atrial and ventricular remodeling findings in the subgroup of patients with the indeterminate form of CD could be due to the small number of patients in this category. Moreover, the absence of increased incidence of arrhythmias in OSA patients in this study should be analyzed with caution, as 24 h Holter monitoring could not detect intermittent arrhythmias. New studies with technologies that analyze long periods of time are warranted.

## Conclusion

OSA is common and independently associated with atrial and ventricular remodeling in patients with CD. The improvement in OSA recognition and treatment may contribute to reducing the morbidity attributed to CD.
